# Mitogenomics of the Olive Seed Weevil, *Anchonocranus oleae* Marshall and Implications for Its Phylogenetic Position in Curculionidae

**DOI:** 10.3390/insects13070607

**Published:** 2022-07-06

**Authors:** Samuel J. Smit, Elleunorah Allsopp, Zwannda Nethavhani, Virgilio Caleca, Rolf G. Oberprieler, Barbara van Asch

**Affiliations:** 1 Department of Genetics, Stellenbosch University, Private Bag X1, Matieland 6702, Stellenbosch 7600, South Africa; cobus.smit@york.ac.uk; 2 Centre for Novel Agricultural Products, Department of Biology, University of York, York YO10 5DD, UK; 20998880@sun.ac.za; 3 Agricultural Research Council, Infruitec-Nietvoorbij, Stellenbosch 7599, South Africa; allsoppe@arc.agric.za; 4 Department of Agricultural, Food and Forest Sciences, University of Palermo, Viale delle Scienze, Edificio 5, 90128 Palermo, Italy; virgilio.caleca@unipa.it; 5 CSIRO Australian National Insect Collection, G.P.O. Box 1700, Canberra, ACT 2601, Australia; curculio@homemail.com.au

**Keywords:** African Wild Olive, *Olea europaea* subsp. *europaea*, *O. europaea* subsp. *cuspidata*, mitochondrial phylogeny

## Abstract

**Simple Summary:**

*Anchonocranus oleae* is a southern African weevil that feeds on the seeds of the African Wild Olive, a close relative of the European cultivated olive tree. The species is known to occur in the Western Cape of South Africa, the main region of olive production in Southern Africa. We generated reference DNA barcodes and the complete mitogenome of *A. oleae* as part of our ongoing genetic cataloguing of insects associated with wild and cultivated olives in South Africa. The phylogenetic position of *A. oleae* in the family Curculionidae was inferred to be in the Curculioninae, Conoderinae, Cossoninae, Molytinae, and Scolytinae (CCCMS) clade but could not be precisely determined due to the paucity of genetic data for adequate taxonomic context, highlighting the need for further coverage of related tribes and genera. Nevertheless, the data generated in this study contribute to the enrichment of baseline information on olive-associated insects, in general, and on the genus *Anchonocranus*, in particular.

**Abstract:**

*Anchonocranus oleae* Marshall (Coleoptera: Curculionidae) is a seed-feeding weevil native to southern Africa; its larvae are known to develop in the fruits of the African Wild Olive and, more rarely, cultivated olives. The species has been mainly found in the Western Cape province of South Africa, but it has remained in relative obscurity because it does not seem to represent a current threat to commercial olive production. As part of an ongoing effort to produce baseline genetic data for olive-associated entomofauna in South Africa, we generated reference DNA barcodes for *A. oleae* collected from wild and cultivated olives and sequenced its mitogenome for assessment of the phylogenetic position of the species in the family Curculionidae. The mitochondrial phylogeny estimate indicated that *A. oleae* shares a common ancestor with *Elaidobius* (tribe Derelomini), but a definite and close relationship to this tribe and the precise tribal placement of *A. oleae* in the subfamily Curculioninae could not be inferred due to the lack of representative mitogenomes of other relevant curculionine tribes and genera. This study will assist future work on the DNA-based species identification, genetic diversity, and phylogenetic position of the genus *Anchonocranus* and related taxa.

## 1. Introduction

Insects occurring on Oleaceae have elicited interest from entomologists for more than a century, mainly in the hope of finding biocontrol agents for the Olive Fruit Fly, *Bactrocera oleae* (Rossi), a major pest of cultivated olives (*Olea europaea* subsp. *europaea*). Surveys of olive-associated entomofauna have mostly been conducted in sub-Saharan Africa and Asia and focus on the discovery and cataloguing of parasitoid wasps [1,2,3,4], as *B. oleae* lacks specialized natural enemies in the ancestral olive-growing Mediterranean region and in its expanded North American range [5].

*Anchonocranus oleae* was described as a new genus and species based on two specimens bred from fruits of the African Wild Olive (*Olea europaea* subsp. *cuspidata*) in South Africa [6], and its larva was subsequently described from the same host (given as *O. verrucosa*) [7]. A similar specimen reared from a seed of *O. chrysophylla* (now also deemed to be *O. europaea* subsp. *cuspidata*) in Eritrea in East Africa was described as *A. oleae* var. *pallida* [sic] [7], but the pale colour for which it was named may only be due to a teneral state. If *A. oleae pallidus* is indeed only a form (or subspecies) of *A. oleae* from South Africa, the geographical distribution of *A. oleae* seems to concord with that of the African Wild Olive, as is the case with other insects associated with Oleaceae in sub-Saharan Africa, including several species of olive fruit flies, olive lace bugs, and olive flea beetles and a diversity of parasitoid, hyperparasitoid, and olive seed wasps [8,9,10,11,12,13]. However, general surveys of insects in the fruits of Oleaceae in Kenya [8] and the African Wild Olive in South Africa [3,9] did not detect the presence of *A. oleae*, and it was only recently again reported from fruits of the African Wild Olive in the Western Cape and Eastern Cape provinces of South Africa, at a very low frequency [14]. Although this paper claimed the rediscovery of the weevil after a century, the species had in fact been recollected in South Africa at regular intervals since its description in the Western Cape in 1927, 1928, 1964, 1978, 1984, and 2003 and in Gauteng (Pretoria) in 2003 and 2020 (based on specimens preserved in the Iziko Museum in Cape Town, the University of Naples in Portici, Italy, and the South African National Collection in Pretoria. Furthermore, in the South African National Collection, there are specimens of apparently different (undescribed) species of *Anchonocranus* collected in the Western Cape in the Knysna area in 1976 and 1981, in the Eastern Cape province at King William’s Town in 1958 and Umtiza in 1988, in the Free State province near Bethulie in 1987, in the North West province at Rustenburg in 1989, and in the Limpopo province at Blouberg in 1987 (*R. Stals*, pers. comm.). From these records, it is evident that *Anchonocranus* is widespread in South Africa and not particularly rare, but the taxonomy of the genus requires comprehensive revision before the taxonomic limits and exact distribution range of *A. oleae* can be established.

The taxonomic and phylogenetic position of *Anchonocranus* is also unclear. It was originally considered to belong in the subfamily Erirhininae and compared with the tribe Storeini [6], in which it was subsequently listed [15,16]. Later, it was compared with the Madagascan genus *Lepidoops* Hustache, with which it shares the unusual feature of having the eyes sparsely covered with scale-like setae and which is classified in the tribe Anthonomini [16,17]. The subfamily Erirhininae is now restricted to more primitive taxa of Curculionidae (with the pedotectal type of male genitalia) and not applicable to *Anchonocranus*, and both Anthonomini and Storeini are treated as tribes of the subfamily Curculioninae [18]. In accordance with a recent reconstitution of the Storeini (restricted to genera in the Australo-Pacific region) [19], *Anchonocranus* was excluded from this tribe and tentatively affiliated with the African *Phacellopterus* group [18], which is also classified in Anthonomini but forms a distinct Afrotropical element [20] that is not evidently closely related to the typical, Palearctic members of the tribe [21]. However, apart from its protruding eyes (the head constricted behind them), *Anchonocranus* shares no specific characters with the *Phacellopterus* group or with typical Anthonomini, differing most prominently in its setose eyes, simple divaricate tarsal claws, and its peculiar flat, shiny scales, making it an apparently isolated genus in the subfamily Curculioninae [18].

The advent of next-generation sequencing techniques expedited the availability of insect mitogenomes, which now represent the most extensively studied genomic material in the order. Insect mitogenomes are commonly used as a source of information for phylogenetic reconstruction, and the results have seldom been drastically incongruent with those derived from morphology or nuclear-gene data [22]. Therefore, the utility of insect mitogenomes for the inference of relationships is widely acknowledged and has contributed to clarifying relationships among Curculionidae [23]. This work reports on the mitochondrial genome of *A. oleae* and presents an assessment of its phylogenetic relationship with other weevils, as part of an ongoing effort to catalogue the genetic diversity of the olive-associated entomofauna in South Africa.

## 2. Materials and Methods

### 2.1. Specimen Collection and Morphological Identification

One *A. oleae* adult emerged in the laboratory in April 2010 from a fruit of *O. e.* subsp. *cuspidata* collected in Stellenbosch in the same month, and one male and one female adult collected on *O. e.* subsp. *cuspidata* fruits and trees in Stellenbosch in January 2014 were identified as *A. oleae* by E. Colonnelli (Rome, Italy) by comparison with photographs provided by V. Caleca of a syntype specimen of *A. oleae* preserved in the Natural History Museum, London, United Kingdom. Although this specimen is labelled as holotype, it is only a syntype, as no holotype was designated in the original description.

Specimens in the South African National Collection in Pretoria were identified by R. Oberprieler in 1987 by comparison with Marshall’s second syntype of *A. oleae*, housed in the Iziko Museum in Cape Town, and in 2020 by R. Stals, and specimens in the University of Naples in Portici were identified by E. Colonnelli in 2018 by comparison with the syntype of *A. oleae* in the Natural History Museum in London. The material in Portici consists of eight specimens, evidently forming part of the same series as the two syntypes (but not seen by Marshall and thus not constituting syntypes), which were collected in Wellington, seemingly by C. P. Lounsbury, and another series of 80 specimens were collected in Wellington in 1928.

**Figure 1:**
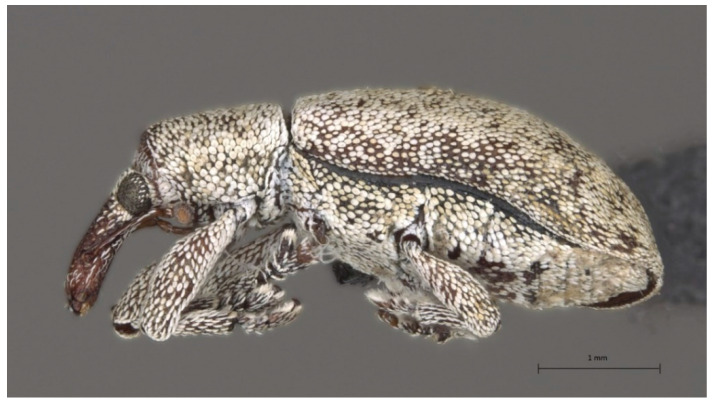
*Anchonocranus oleae* specimen collected in Grahamstown and deposited in the Iziko Museum South Africa (Cape Town), assigned with the coden SAM-COL-A082796.

The adults and larvae used in this study for DNA analyses, imaging, and museum deposit were collected from the fruits of the African Wild Olive and from cultivated olives in the Western Cape province of South Africa between February 2016 and March 2018 during a study on olive seed wasps [24] and were identified by V. Caleca (Table 1). Total DNA was individually extracted from whole larvae and from one leg of an adult using a standard phenol-chloroform method [25] and was stored at −20 °C until downstream analyses. One adult specimen was photographed and deposited in the insect collection of the Iziko Museums of South Africa in Cape Town with the coden SAM-COL-A082796 (Figure 1).

### 2.2. DNA Barcoding of Anchonocranus oleae

Twenty-three specimens were sequenced for the standard COI barcoding region using the universal arthropod primer pair LCO1490/HCO2198 [26]. PCR amplifications were performed in a total volume of 5 μL, containing 1× of QIAGEN Multiplex PCR Kit (QIAGEN), 0.2 μM of each primer, 0.5 μL of MilliQ H_2_O, and 1.0 μL of template DNA. The thermal cycling program consisted of 95 °C for 15 min; 35 cycles of 94 °C for 30 s, 54 °C for 90 s, 72 °C for 90 s, and 72 °C for 10 min. PCR products were sequenced in both directions using the BigDye Terminator v3.1 Cycle Sequencing Kit (Applied Biosystems) at the Central Analytical Facilities of Stellenbosch University, South Africa. The sequences were aligned using the MAFFT algorithm [27] in Geneious Prime v2020.1.2 (https://www.geneious.com; accessed on 12 December 2021). Intraspecific maximum pairwise distance (max p-distance) was calculated in MEGA X v10.1 [28] under the Kimura 2-parameter (K2P) model [29]. A haplotype median-joining network of the COI sequences was constructed using the software Network 10 (www.fluxus-engineering.com; accessed on 12 December 2021) [30]. The new DNA barcodes were deposited on GenBank under the accession numbers ON504300-ON504321.

### 2.3. Mitogenome Sequencing: Assembly and Annotation

Total DNA from one adult specimen was sequenced using the Ion Torrent ProtonTM sequencing platform (ThermoFisher Scientific, Waltham, MA, USA) at the Central Analytical Facilities of Stellenbosch University, South Africa. Sequence libraries were prepared using the NEXTflex™ DNA Sequencing Kit for Ion Platforms (PerkinElmer, Waltham, MA, USA) according to the BI00 Scientific v15.12 protocol. Libraries were pooled and sequenced using the Ion PI HiQ™ Sequencing Solutions Kit (Life Technologies, CA, USA). Reads were mapped to the mitogenome of *Anthonomus eugenii* (Curculioninae) (GenBank accession MK654676.1) in Geneious Prime with the default mapping parameters for the medium sensitivity/fast option. The consensus sequence was annotated in parallel using the MITOS Web Server (http://mitos.bioinf.uni-leipzig.de/index.py; accessed on 20 December 2021) [31] and the ARWEN software (http://130.235.244.92/ARWEN/; accessed on 20 December 2021) [32], using the invertebrate mitochondrial genetic code. Annotations were manually curated to ensure that the size and position of protein-coding genes (PCGs) were congruent with those of other Curculionidae. The positions of the transfer RNA genes (tRNAs) predicted by MITOS and ARWEN were compared, and the most probable were manually selected. The positions of the ribosomal RNA genes (rRNAs) were determined with MITOS and manually curated by comparison with those of other Curculionidae. The large non-coding region between the 12 s rRNA gene and the Q-M-ND2 gene cluster was annotated as the AT-rich (control) region. Nucleotide composition and compositional biases [AT-skew = (A − T)/(A + T); CG-skew = (G − C)/(G + C)] were calculated using Geneious Prime. Indices of codon usage bias were calculated in DnaSP6 (http://www.ub.edu/dnasp/; accessed on 20 December 2021) [33] using the *Drosophila melanogaster* mitochondrial genetic code.

### 2.4. Phylogenetic Analysis

The phylogenetic position of *A. oleae* in Curculionidae was inferred in the context of the mitogenomes of 74 other species representing 11 subfamilies across 50 tribes, with *Anoplophora glabripennis* (Cerambycidae) and *Crioceris duodecimpunctata* (Chrysomelidae) as outgroups [23,34,35,36,37,38,39,40,41,42,43,44,45,46,47,48,49,50,51,52,53,54,55,56,57] (Table S1). Among the numerous weevil mitogenomes available on GenBank, these were selected because they represent the greatest available diversity of potentially related taxa in the Curculioninae, Conoderinae, Cossoninae, Molytinae, and Scolytinae (CCCMS) clade of the family Curculionidae [58], in which *Anchonocranus* evidently belongs, in particular of tribes in the current subfamily Curculioninae, in which it has been traditionally classified.

Translation alignments were performed for all protein-coding genes (PCG) separately using the MAFFT algorithm in Geneious Prime except ND2, which was excluded from the dataset because the gene was not sequenced in many of the mitogenomes available on GenBank. Stop codons and alignment gaps were removed manually, and individual PCG alignments were concatenated to produce a single nucleotide sequence alignment. Maximum Likelihood (ML) trees were inferred using IQ-Tree 2 [59] on the Viking Cluster, a high-performance computer facility available at the University of York. Model selection was automatically determined [60] with partitioned analyses by gene [61], using 1000 replicates for both Ultra-Fast Bootstrap [62] and Sh-aLRT support [63] (Table S2). The final tree was drawn using FigTree v1.4.4. (http://tree.bio.ed.ac.uk/software/figtree/; accessed on 10 February 2022). The mitogenome of *A. oleae* was deposited on GenBank under the accession number ON859837.

## 3. Results and Discussion

*Anchonocranus oleae* is part of the rich assemblage of native sub-Saharan African insects associated with wild and cultivated olive trees and fruits. Part of the diversity of parasitoid, hyperparasitoid and seed wasps, olive fruit flies, and olive lace bugs found in South Africa has been recently characterized at the DNA sequence level, either with complete mitogenomes [10,11], DNA barcodes [64], or other genetic data that have allowed for new insights into the diversity and lifestyles of olive insects [12]. This work further contributes to the genetic cataloguing of the entomofauna of *O. europaea* in southern Africa.

### 3.1. DNA Barcoding of Anchonocranus oleae

**Figure 2:**
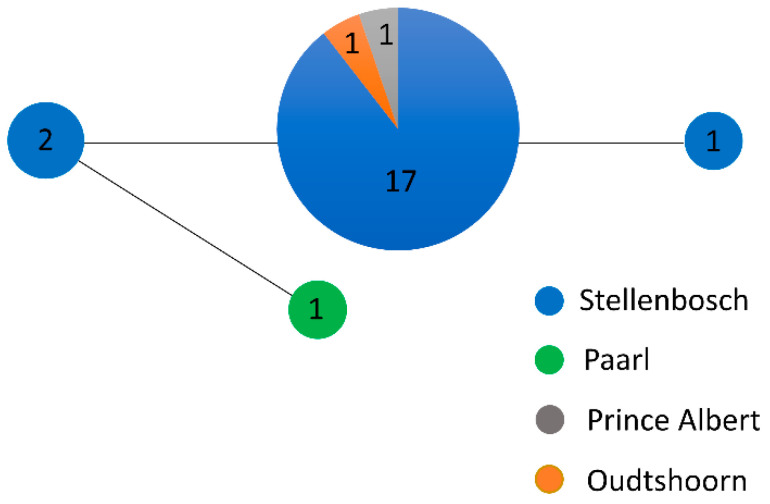
Median-joining network of four haplotypes found in 23 specimens of *Anchonocranus oleae* at four areas in the Western Cape of South Africa. The circles are proportional to the frequency of haplotypes, and the length of the lines represents one mutational step between the haplotypes.

The 23 specimens analysed at the DNA sequence level were found at four sites in the Western Cape and had four closely related haplotypes with low intraspecific max p-distance (0.44%) (Figure 2). These results support the conspecificity of the specimens and will be useful for comparing genetic data for other apparent species of *Anchonocranus.*

### 3.2. The Mitochondrial Genome of Anchonocranus oleae

**Figure 3:**
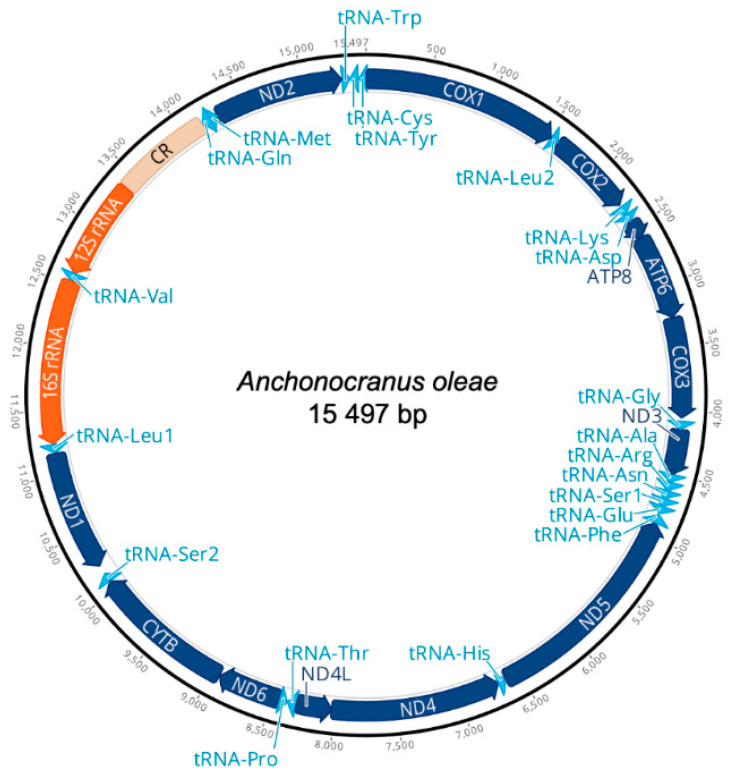
Mitochondrial genome organization of the Olive Seed Weevil, *Anchonocranus oleae*. The arrows represent the direction of the genes.

The Ion Torrent run generated a total of 15,006,094 reads with an average size of 170 bp, of which 6533 reads were mapped to the reference mitogenome with an average coverage of 69X. The final consensus sequence was 14,725 bp long (excluding the AT-rich region), in line with the range found in other Curculionidae [34]. The common metazoan complement of 13 PCGs and two rRNAs was identified, as well as the non-coding (AT-rich) region containing the control for mitogenomic replication and transcription (Table 2; Figure 3). The mitogenome of *A. oleae* is compact, with a total of 126 intergenic nucleotides distributed among eight regions, of which the largest (70 bp) is located between tRNA^Ser2^ and ND1. The AT-rich region (772 bp) is located between 12 S rRNA and tRNA^Gln^. The general organization and transcription orientation of PCGs are identical to the hypothetical ancestral Arthropoda PCGs [65], of which nine are encoded in the majority (J) strand and four in the minority (N) strand. Four different codons initiated the translation of PCGs: ATT (COI, COII, ATP8, and ND5), ATG (COIII, ND4, ND4L, and ND2), ATA (ATP6, ND3, and ND6) and TTG (ND1).

The total sequence had the high A + T content (76.29%) typically found in insects, varying from 68.18% in COI to 84.62% in ATP8, in line with previous results in the family [23] (Table 3). The complete set of PCGs had a C + G content of 29.95% across all codon positions, 31.6% at the second-codon position and 12.90% at the third-codon position. The difference in nucleotide composition resulted in a skew of G over C on the N-strand (GC-skew = 0.30) and C over G on the J-strand (GC-skew = −0.18). AT-skew showed a higher proportion of T over A on the N-strand (−0.22) and less substantially on the J-strand (−0.08).

**Figure 4:**
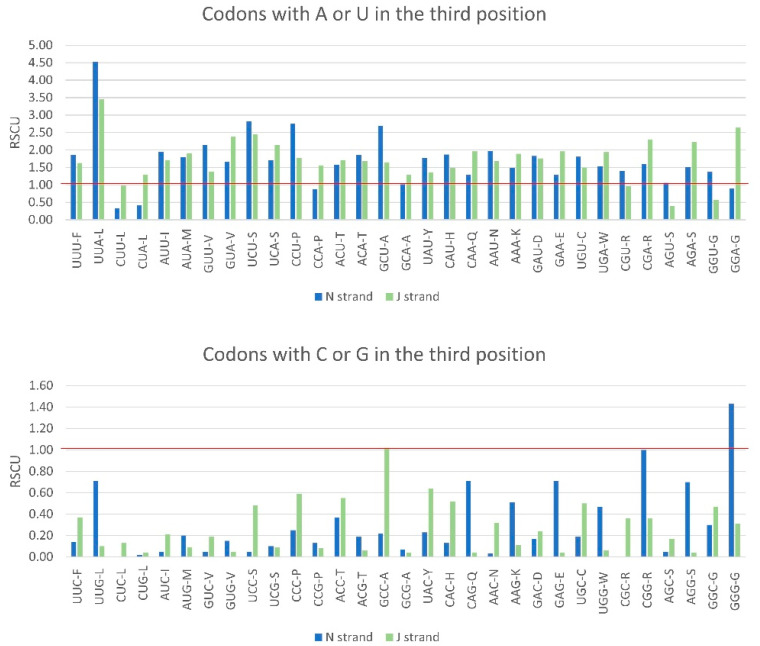
Codon usage and relative synonymous codon usage (RSCU) in the mitogenome of *Anchonocranus oleae*.

Relative synonymous codon usage (RSCU) is the ratio of the observed frequency of a codon by its expected frequency under the assumption of equal codon usage [66]. RSCU values greater than 1.0 indicate that the corresponding codons are used more frequently than the expected frequency, whereas the reverse is true for RSCU values less than 1.0. The concatenated PCGs of *A. oleae* had all 62 sense codons, and 27 codons (44%) had a higher frequency than expected by chance (RSCU > 1), of which the most frequent was UUA (RSCU = 3.93) (Table 4). A total of 35 codons (56%) had a lower frequency than expected (RSCU < 1), of which 31 had G or C at the third position, and the least-used codon was CUG (RSCU 0.03). All high-frequency codons had A or T at the third position, and all codons with very low frequency (RSCU < 0.6) had G or C at the third position. When considering codons ending with A or T (AT3 codons) and codons ending with C or G (CG3 codons) separately in N-strand genes and in J-strand genes, it was apparent that AT3 codons were more frequent than expected and CG3 were less frequent than expected, independently of the strand (Figure 4). This result was also evident in the values of other measures for codon usage bias when considering all PCGs and PCGs on the J-strand and on the N-strand separately. The effective number of codons (ENC) is used to measure the bias of synonymous codons and varies between 20 (only one codon is used for each amino acid) and 61 (when codons are used randomly) [67]. The ENC of the total PCGS was 37.96, indicating a moderate level of codon usage bias. The codon bias index (CBI) is also a measure of the deviation from the equal use of synonymous codons. CBI values range from 0 (uniform use of synonymous codons) to 1 (only preferred codons used). The CBI of *A. oleae* was 0.675, indicating strong bias toward the use of a subset of optimal codons. Therefore, codon usage bias in the mitogenome of *A. oleae* seems to be the result of its nucleotide composition bias towards high A + T content and not of the strand (J or N) where the genes are encoded.

All expected tRNAs were identified and annotated in the mitogenome sequence of *A. oleae*, except tRNA^Ile^. This gene was also not identified in other Curculionidae species included in this study, as noted in numerous previous works [34,36,38,50,55,68,69]. The difficulty in identifying tRNA^Ile^ most likely stems from its location, as the gene has been found within a large non-coding region, either unannotated or annotated as the control region.

### 3.3. Phylogenetic Position of Anchonocranus oleae

**Figure 5:**
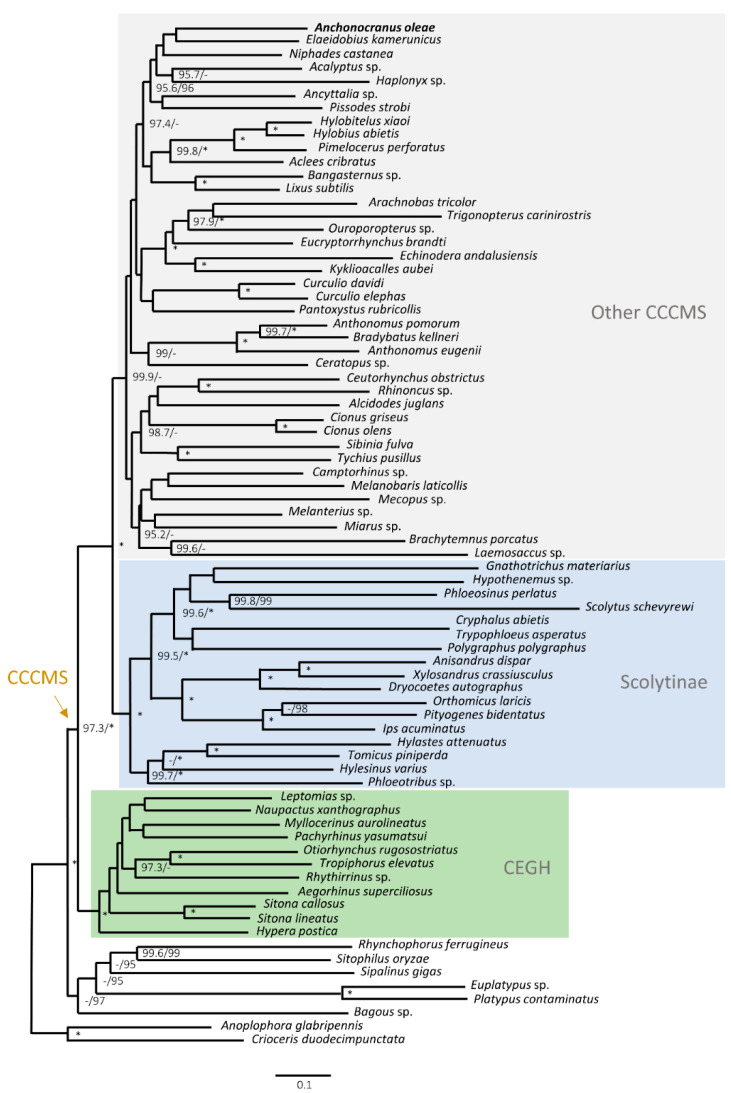
Phylogenetic position of *Anchonocranus oleae* among 73 other species of Curculionidae, with *Anoplophora glabripennis* (Cerambycidae) and *Crioceris duodecimpunctata* (Chrysomelidae) as outgroups. The tree was constructed using maximum likelihood based on a nucleotide alignment of 12 mitochondrial protein-coding genes. Node support values: SH-aLRT (%)/ultrafast bootstrap (%); values < 95% are not shown or represented by a hyphen (-); values of 100% are represented by an asterisk (*).

The phylogenetic position of *A. oleae* in Curculionidae was explored by ML analysis, using 74 other selected mitogenomes available for the family on GenBank (Figure 5). The broad groups recovered were largely compatible with those of larger samples of weevil mitogenomes [23,70], as well as those recovered in a recent analysis of a large dataset of nuclear protein-coding genes [71]. Four main features were recovered: (a) the sister-group relationship of the subfamilies Dryophthorinae and Platypodinae and their basal position in the family (outside of the “higher” Curculionidae), (b) the basal position of the subfamily Bagoinae (also outside of the “higher” Curculionidae), (c) the basal division of the “higher” Curculionidae into two monophyletic groups, termed CEGH (subfamilies Cyclominae, Entiminae, Gonipterini, and Hyperinae) and CCCMS (Conoderinae, Cossoninae, Curculioninae, Molytinae, and Scolytinae) [58], and (d) in the CCCMS clade a basal division between the subfamily “Scolytinae” and the remainder of all taxa. Support for the monophyly of the remaining CCCMS taxa was also high, but most relationships among these taxa were poorly supported and could not identify meaningful clades at the current subfamily of the tribal level. *Anchonocranus* was included in this remainder of CCCMS taxa and clustered together with the genera *Elaidobius* (tribe Derelomini), *Acalyptus* (Acalyptini), *Ancyttalia* (Eugnomini), *Haplonyx* (Cryptoplini), *Niphades* (Aminyopini), and *Pissodes* (Pissodini), the former four currently classified in the subfamily Curculioninae but the latter two in Molytinae. The affiliation of *Anchonocranus* was with *Elaidobius* with nodal support of 78/58 in ML-PCG123, which is the only African genus of the cluster and, similar to the other taxa of Derelomini (and also most Acalyptini), associated with palms. No affiliation was revealed with the tribe Anthonomini (*Anthonomus* and *Bradybatus*). In the absence of other relevant African genera being included in the analysis, such as the *Phacellopterus* group and the Madagascan *Lepidoops* but also other seed-feeding taxa, our analysis only revealed that *Anchonocranus* belongs in the CCCMS clade of Curculionidae, and possibly in the subfamily Curculioninae, but its exact phylogenetic position remains unresolved and its classification as Curculioninae *incertae sedis* [18] is appropriate for now.

### 3.4. Notes on Host Plants of Anchonocranus oleae

All the specimens analysed in this study were collected from fruits of the European Olive (*O. europaea* subsp. *europaea*) and African Wild Olive (*O. europaea* subsp. *cuspidata*). In our previous survey of these trees, *A. oleae* was found at a very low frequency compared to other insects known to be exclusively associated with fruits of *O. e. europaea* and *O. e. cuspidata* in South Africa [64]. However, we did not survey other species of Oleaceae in addition to *O. europaea*. Specimens of *A. oleae* in the South African National Collection in Pretoria with host data were also mostly collected or reared from *O. e. cuspidata,* except for a series of five specimens collected by co-author R. Oberprieler in 1984 on *O. capensis* at Michell’s Pass near Ceres in the Western Cape province. This record indicates that *A. oleae* may also breed in the fruits of *O. capensis* and perhaps other *Olea* species. Furthermore, the existence of other *Anchonocranus* species in South Africa (and possibly elsewhere) indicates that the fruits of other *Olea* species (such as *O. exasperata* and *O. woodiana* in South Africa) may also serve as hosts for *Anchonocranus* species. Extensive fieldwork is required to explore this possibility. Clarification of the natural hostplants of *A. oleae* stands to have implications for the biocontrol of the African Wild Olive in other countries, e.g., Australia, should the weevil be considered in this regard.

## Figures and Tables

**Table 1:** List of specimens of *Anchonocranus oleae* (Coleoptera: Curculionidae), used for DNA analyses (barcoding and sequencing of complete mitochondrial genome) and photographic imaging and deposition in the insect collection of the Iziko Museums of South Africa (Cape Town). Cultivated host—*Olea europaea* subsp. *europaea*; wild host—*Olea europaea* subsp. *cuspidata.*

Specimen	Life Stage	Collection Date	Region	Latitude	Longitude	Olive Host	Use
W01	Larva	24 February 2016	Stellenbosch	−33.99514287	18.870638997	Cultivated	DNA barcode
W12	Larva	15 July 2016	Stellenbosch	−33.91391193	18.860714412	Cultivated	DNA barcode
W13	Larva	15 July 2016	Stellenbosch	−33.91391193	18.860714412	Cultivated	DNA barcode
W14	Larva	15 July 2016	Stellenbosch	−33.91391193	18.860714412	Cultivated	DNA barcode
W15	Larva	15 July 2016	Stellenbosch	−33.91391193	18.860714412	Cultivated	DNA barcode
W16	Larva	15 July 2016	Stellenbosch	−33.91391193	18.860714412	Cultivated	DNA barcode
WI	Larva	1 March 2017	Stellenbosch	−33.99514287	18.870638997	Cultivated	DNA barcode
WII	Larva	1 March 2017	Stellenbosch	−33.99514287	18.870638997	Cultivated	DNA barcode
WIII	Larva	1 March 2017	Stellenbosch	−33.99514287	18.870638997	Cultivated	DNA barcode
WVI	Larva	8 March 2017	Oudtshoorn	−33.49409606	22.494753782	Wild	DNA barcode
W27	Adult	28 April 2016	Grahamstown	−33.31910297	26.518800775	Wild	Museum deposit/photo
W29	Larva	29 March 2017	Paarl	−33.68018382	18.907568940	Cultivated	DNA barcode
W30	Larva	4 November 2017	Stellenbosch	−33.99514287	18.870638997	Cultivated	DNA barcode
W31	Larva	4 November 2017	Stellenbosch	−33.99514287	18.870638997	Cultivated	DNA barcode
W32	Larva	4 November 2017	Stellenbosch	−33.99514287	18.870638997	Cultivated	DNA barcode
W33	Larva	4 November 2017	Stellenbosch	−33.99514287	18.870638997	Cultivated	DNA barcode
W34	Larva	4 April 2017	Stellenbosch	−33.99514287	18.870638997	Cultivated	DNA barcode
W35	Larva	15 July 2016	Stellenbosch	−33.91391193	18.860714412	Wild	DNA barcode
W37	Larva	15 July 2016	Stellenbosch	−33.91391193	18.860714412	Wild	DNA barcode
W38	Larva	15 July 2016	Stellenbosch	−33.91391193	18.860714412	Wild	DNA barcode
W39	Larva	15 July 2016	Stellenbosch	−33.91391193	18.860714412	Wild	DNA barcode
W40	Larva	15 July 2016	Stellenbosch	−33.91391193	18.860714412	Wild	DNA barcode
W41	Larva	7 March 2017	Prince Albert	−33.30853418	22.526805331	Cultivated	DNA barcode
W47	Adult	13 March 2018	Stellenbosch	−33.99514287	18.870638997	Cultivated	Mitogenome

**Table 2:** List of specimens of *Anchonocranus oleae* (Coleoptera: Curculionidae) used for DNA analyses (barcoding and sequencing of complete mitochondrial genome) and photographic imaging and deposition in the insect collection of the Iziko Museum of South Africa (Cape Town). Cultivated host—*Olea europaea* subsp. *europaea*; wild host—*Olea europaea* subsp. *cuspidata.*

Gene/Region	Code	Coordinates	Strand	Size (bp)	Anticodon	Start	Stop	IGN
COI	-	1–1540	J	1540	-	ATT	T--	−8
tRNA^Leu2^	L2	1541–1606	J	66	TAA	-	-	0
COII	-	1607–2290	J	684	-	ATT	TAA	0
tRNA^Lys^	K	2307–2378	J	72	CTT	-	-	16
tRNA^Asp^	D	2378–2448	J	66	GTC	-	-	−1
ATP8	-	2444–2599	J	156	-	ATT	TAG	0
ATP6	-	2596–3266	J	671	-	ATA	TA-	−4
COIII	-	3267–4055	J	789	-	ATG	TAA	0
tRNA^Gly^	G	4076–4141	J	66	TCC	-	-	20
ND3	-	4142–4495	J	354	-	ATA	TAA	0
tRNA^Ala^	A	4498–4562	J	65	TGC	-	-	2
tRNA^Arg^	R	4563–4631	J	69	TCG	-	-	0
tRNA^Asn^	N	4629–4691	J	63	GTT	-	-	−3
tRNA^Ser1^	S1	4692–4757	J	66	AGA	-	-	0
tRNA^Glu^	E	4758–4821	J	64	TTC	-	-	0
tRNA^Phe^	F	4831–4897	N	67	GAA	-	-	9
ND5	-	4901–6619	N	1719	-	ATT	TAA	3
tRNA^His^	H	6620–6684	N	65	GTG	-	-	0
ND4	-	6687–8018	N	1332	-	ATG	TAG	2
ND4L	-	8012–8296	N	285	-	ATG	TAA	−7
tRNA^Thr^	T	8299–8366	J	68	TGT	-	-	2
tRNA^Pro^	P	8367–8432	N	66	TGG	-	-	0
ND6	-	8433–8935	J	503	-	ATA	TA-	0
CYTB	-	8936–10075	J	1140	-	ATG	TAA	0
tRNA^Ser2^	S2	10,079–10,147	J	69	TGA	-	-	3
ND1	-	10,218–11,168	N	951	-	TTG	TAG	70
tRNA^Leu1^	L1	11,169–11,235	N	67	TAG	-	-	0
16s rRNA	-	11,236–12,534	N	1299	-	-	-	0
tRNA^Val^	-	12,535–12,599	N	65	TAC	-	-	0
12s rRNA	-	12,600–13,378	N	779	-	-	-	0
AT-rich region	-	13,380–14,151	-	772	-	-	-	0
tRNA^Ile^	I	n.d.	n.d.	n.d.	n.d.	-	-	n.d.
tRNA^Gln^	Q	14,154–14,224	N	71	TTG	-	-	0
tRNA^Met^	M	14,222–14,291	J	70	CAT	-	-	−3
ND2	-	14,292–15,305	J	1014	-	ATG	TAA	0
tRNA^Trp^	W	15,308–15,373	J	66	TCA	-	-	2
tRNA^Cys^	C	15,375–15,436	N	62	GCA	-	-	1
tRNA^Tyr^	Y	15,442–15,497	N	64	GTA	-	-	5

**Table 3:** Nucleotide composition of the complete mitochondrial sequence of the Olive Seed Weevil, *Anchonocranus oleae*. AT-skew = (A − T)/(A + T); CG-skew = (G − C)/(G + C).

Gene/Region	Strand	A%	C%	G%	T%	A + T%	G + C%	AT-Skew	GC-Skew	Size (bp)	% of Total bp
COI	J	32.14	17.86	13.96	36.04	68.18	31.82	−0.06	−0.12	1540	9.94
COII	J	35.67	16.37	10.23	37.72	73.39	26.61	−0.03	−0.23	684	4.41
COIII	J	32.95	16.60	12.17	38.28	71.23	28.77	−0.08	−0.15	789	5.09
CYTB	J	31.58	15.00	12.37	41.05	72.63	27.37	−0.13	−0.10	1140	7.36
ATP6	J	32.64	16.39	8.94	42.03	74.66	25.34	−0.13	−0.29	671	4.33
ATP8	J	42.31	11.54	3.85	42.31	84.62	15.38	0.00	−0.57	156	1.01
ND1	N	45.64	16.19	8.73	29.44	75.08	24.92	−0.22	0.30	951	6.14
ND2	J	35.50	14.69	7.99	41.81	77.32	22.68	−0.08	−0.30	1014	6.54
ND3	J	36.44	11.58	8.47	43.50	79.94	20.06	−0.09	−0.15	354	2.28
ND4	N	47.07	13.59	9.31	30.03	77.10	22.90	−0.22	0.18	1332	8.60
ND4L	N	52.63	11.23	5.61	30.53	83.16	16.84	−0.27	0.33	285	1.84
ND5	N	46.48	13.26	9.54	30.72	77.20	22.80	−0.21	0.16	1719	11.09
ND6	J	37.77	10.34	6.56	45.33	83.10	16.90	−0.09	−0.22	503	3.25
PCGs (J)	J	33.83	15.51	10.71	39.95	73.78	26.22	−0.08	−0.18	6828	44.06
PCGs (N)	N	29.43	8.76	16.14	45.68	75.11	24.89	−0.22	0.21	948	6.12
Total PCGs	J + N	32.41	13.02	11.92	42.64	75.05	24.95	−0.14	−0.04	11,103	71.65
16S rRNA	N	42.57	12.93	6.08	38.41	80.99	19.01	0.05	−0.36	1299	8.38
12S rRNA	N	38.38	14.38	7.45	39.79	78.18	21.82	−0.02	−0.32	779	5.03
Total rRNAs	N	41.00	13.47	6.59	38.93	79.93	20.07	0.03	−0.34	2078	13.41
Total tRNAs	J + N	39.93	14.03	9.42	36.62	76.55	23.45	0.04	−0.20	1390	8.97
AT-rich		42.95	10.87	7.89	38.16	81.24	18.76	0.06	−0.16	773	4.99
Mitogenome		39.55	14.34	9.37	36.73	76.29	23.71	0.04	−0.21	15,497	100.00

**Table 4:** Codon usage in the mitochondrial genome of the Olive Seed Weevil, *Anchonocranus oleae*. Amino acids are labelled according to the IUPAC-IUB single letter codes. N—total number of occurrences in all protein-coding genes, RSCU—relative synonymous codon usage.

Codon-AA	Freq	RSCU	Codon-AA	Freq	RSCU	Codon-AA	Freq	RSCU
UUU-F	317	1.72	CCC-P	16	0.50	GAU-D	59	1.76
UUC-F	51	0.28	CCA-P	44	1.39	GAC-D	7	0.21
UUA-L	372	3.93	CCG-P	3	0.09	GAA-E	65	1.69
UUG-L	35	0.37	ACU-T	73	1.68	GAG-E	12	0.31
CUU-L	65	0.69	ACC-T	22	0.51	UGU-C	31	1.68
CUC-L	7	0.07	ACA-T	75	1.72	UGC-C	6	0.32
CUA-L	86	0.91	ACG-T	4	0.09	UGA-W	85	1.81
CUG-L	3	0.03	GCU-A	79	2.01	UGG-W	9	0.19
AUU-I	346	1.77	GCC-A	29	0.74	CGU-R	15	1.13
AUC-I	44	0.23	GCA-A	47	1.20	CGC-R	3	0.23
AUA-M	257	1.86	GCG-A	2	0.05	CGA-R	27	2.04
AUG-M	19	0.14	UAU-Y	141	1.56	CGG-R	8	0.60
GUU-V	73	1.76	UAC-Y	40	0.44	AGU-S	30	0.70
GUC-V	5	0.12	CAU-H	54	1.57	AGC-S	5	0.12
GUA-V	84	2.02	CAC-H	15	0.43	AGA-S	81	1.89
GUG-V	4	0.10	CAA-Q	57	1.78	AGG-S	15	0.35
UCU-S	112	2.62	CAG-Q	7	0.22	GGU-G	40	0.86
UCC-S	12	0.28	AAU-N	169	1.77	GGC-G	19	0.41
UCA-S	83	1.94	AAC-N	22	0.23	GGA-G	93	2.01
UCG-S	4	0.09	AAA-K	101	1.74	GGG-G	33	0.71
CCU-P	64	2.02	AAG-K	15	0.26			

## Data Availability

DNA barcode sequences are available on GenBank (accession numbers ON504300-ON504321), as well as the complete mitochondrial genome (accession number ON859837.

## Supplementary Materials

The following supporting information can be downloaded at: https://www.mdpi.com/article/10.3390/insects13070607/s1, Table S1: Mitogenomes in phylogeny. Table S2: Best-fit evolutionary models.
