# Supplementary material for: Mitogenomics of the Olive Seed Weevil, Anchonocranus oleae Marshall and Implications for Its Phylogenetic Position in Curculionidae
Source: Insects. 2022 Jul 6;13(7):607. doi: 10.3390/insects13070607 (PMC9321040; doi:10.3390/insects13070607)
Supplement: Supplementary file 1 [file insects-13-00607-s001.zip › insects-1698492-supplementary.pdf]

Table S1. List of the 77 mitochondrial sequences used in the phylogenetic reconstruction of the family Curculionidae.

Family	Subfamily	Tribe	Species	GenBank accession	Reference
Curculionidae	Dryophthorinae	Rhynchophorini	*Sitophilus oryzae*	NC_030765.1	[56]
Curculionidae	Cyclominae	Aterpini	*Aegorhinus superciliosus*	NC_027577.1	[34]
Curculionidae	Entiminae	Tanymecini	*Leptomias* sp.	MT536938.1	[43]
Curculionidae	Dryophthorinae	Rhynchophorini	*Rhynchophorus ferrugineus*	KT428893.1	[47]
Curculionidae	Molytinae	Hylobiini	*Pimelocerus perforatus*	NC_053826.1	[57]
Curculionidae	Conoderinae	Mecopini	*Mecopus* sp.	MH431911.1	Unpublished
Curculionidae	Curculioninae	Anthonomini	*Bradybatus kellneri*	KX087247.1	Unpublished
Curculionidae	Curculioninae	Curculionini	*Curculio elephas*	KX087269.1	Unpublished
Curculionidae	Curculioninae	Cryptoplini	*Haplonyx* sp.	MH431909.1	Unpublished
Curculionidae	Curculioninae	"Cleogonini"	*Pantoxystus rubricollis*	KX087330.1	Unpublished
Curculionidae	Dryophthorinae	Orthognathini	*Sipalinus gigas*	NC_053351.1	Unpublished
Curculionidae	Entiminae	Byrsopagini	*Tropiphorus elevatus*	KX087368.1	Unpublished
Curculionidae	Molytinae	Cryptorhynchini	*Arachnobas tricolor*	KX087241.1	Unpublished
Curculionidae	Molytinae	Lixini	*Lixus subtilis*	MW413392.1	Unpublished
Curculionidae	Platypodinae	Platypodini	*Euplatypus* sp.	KX035180.1	Unpublished
Curculionidae	Scolytinae	Xyleborini	*Anisandrus dispar*	NC_036293.1	Unpublished
Curculionidae	Scolytinae	Cryphalini	*Cryphalus abietis*	MT410860.1	Unpublished
Curculionidae	Scolytinae	Dryocoetini	*Dryocoetes autographus*	NC_036287.1	Unpublished
Curculionidae	Scolytinae	Corthylini	*Gnathotrichus materiarius*	NC_036294.1	Unpublished
Curculionidae	Scolytinae	Hylastini	*Hylastes attenuatus*	NC_036290.1	Unpublished
Curculionidae	Scolytinae	Cryphalini	*Hypothenemus* sp.	KX035163.1	Unpublished
Curculionidae	Scolytinae	Ipini	*Ips acuminatus*	MK988441.1	Unpublished
Curculionidae	Scolytinae	Ipini	*Orthotomicus laricis*	NC_036291.1	Unpublished
Curculionidae	Scolytinae	Phloeotribini	*Phloeophthorus* sp.	NC_057470.1	Unpublished
Curculionidae	Scolytinae	Phloeosinini	*Phloeosinus perlatus*	KX035210.1	Unpublished
Curculionidae	Scolytinae	Ipini	*Pityogenes bidentatus*	NC_036289.1	Unpublished
Curculionidae	Scolytinae	Polygraphini	*Polygraphus poligraphus*	MN528600.1	Unpublished
Curculionidae	Scolytinae	Cryphalini	*Trypophloeus asperatus*	NC_036285.1	Unpublished
Curculionidae	Scolytinae	Xyleborini	*Xylosandrus crassiusculus*	NC_036284.1	Unpublished
Curculionidae	Bagoinae	Bagoini	*Bagous* sp.	MH404106.1	[23]
Curculionidae	Curculioninae	Acalyptini	*Acalyptus* sp.	MH404107.1	[23]
Curculionidae	Curculioninae	Eugnomini	*Ancyttalia* sp.	MH404121.1	[23]
Curculionidae	Curculioninae	Ceratopodini	*Ceratopus* sp.	MH473535.1	[23]
Curculionidae	Curculioninae	Cionini	*Cionus griseus*	MH404125.1	[23]
Curculionidae	Curculioninae	Cleogonini	*Melanterius* sp.	MH404113.1	[23]
Curculionidae	Curculioninae	Mecinini	*Miarus* sp.	MH404105.1	[23]
Curculionidae	Curculioninae	Ceutorhynchini	*Rhinoncus* sp.	MH404119.1	[23]
Curculionidae	Curculioninae	Tychiini	*Sibinia fulva*	MH404129.1	[23]
Curculionidae	Cyclominae	Rhythirrinini	*Rhythirrinus* sp.	MH404126.1	[23]
Curculionidae	Molytinae	Lixini	*Bangasternus* sp.	MH404135.1	[23]
Curculionidae	Molytinae	Ithyporini	*Camptorhinus* sp.	MH404122.1	[23]
Curculionidae	Molytinae	Mesoptiliini	*Laemosaccus* sp.	MH404116.1	[23]
Curculionidae	Molytinae	Cryptorhynchini	*Ouroporopterus* sp.	MH404099.1	[23]
Curculionidae	Scolytinae	Hylesinini	*Hylesinus varius*	MH281571.1	[23]
Curculionidae	Scolytinae	Hylurgini	*Tomicus piniperda*	MH281570.1	[23]
Curculionidae	Conoderinae	Baridini	*Melanobaris laticollis*	JN163955.1	[41]
Curculionidae	Cossoninae	Onycholipini	*Brachytemnus porcatus*	JN163960.1	[41]
Curculionidae	Curculioninae	Ceutorhynchini	*Ceutorhynchus obstrictus*	JN163956.1	[41]
Curculionidae	Curculioninae	Cionini	*Cionus olens*	JN163958.1	[41]
Curculionidae	Entiminae	Otiorhynchini	*Otiorhynchus rugosostriatus*	JN163969.1	[41]
Curculionidae	Entiminae	Sitonini	*Sitona lineatus*	JN163948.1	[41]
Curculionidae	Molytinae	Hylobiini	*Hylobius abietis*	JN163954.1	[41]
Curculionidae	Molytinae	Cryptorhynchini	*Kyklioacalles aubei*	JN163957.1	[41]

Curculionidae	Entiminae	Sitonini	*Sitona callosus*	MF594624.1	[44]
Curculionidae	Molytinae	Cryptorhynchini	*Trigonopterus carinirostris*	NC_050891.1	[53]
Curculionidae	Curculioninae	Tychiini	*Tychius pusillus*	MK692568.1	[54]
Curculionidae	Hyperinae	Hyperini	*Hypera postica*	MK692605.1	[54]
Curculionidae	Molytinae	Cryptorhynchini	*Echinodera andalusiensis*	MK692645.1	[54]
Chrysomeli-dae*	Criocerinae	Criocerini	*Crioceris duodecimpunctata*	NC003372.1	[52]
Curculionidae	Molytinae	Aminyopini	*Niphades castanea*	MT232762.1	[39]
Curculionidae	Entiminae	Naupactini	*Naupactus xanthographus*	NC_018354.1	[55]
Curculionidae	Scolytinae	Scolytini	*Scolytus schevyrewi*	NC_046589.1	[49]
Curculionidae	Molytinae	Hylobiini	*Hylobitelus xiaoi*	NC_022680.1	[50]
Curculionidae	Molytinae	Mecysolobini	*Alcidodes juglans*	NC_041669.1	[42]
Curculionidae	Platypodinae	Platypodini	*Platypus contaminatus*	NC_045889.1	[40]
Curculionidae	Entiminae	Cyphicerini	*Myllocerinus aurolineatus*	NC_040931.1	[48]
Curculionidae	Curculioninae	Derelomini	*Elaeidobius kamerunicus*	NC_049880.1	[51]
Curculionidae	Entiminae	Polydrusini	*Pachyrhinus yasumatsui*	MF807224.1	[46]
Curculionidae	Curculioninae	Curculionini	*Curculio davidi*	NC_034293.1	[38]
Curculionidae	Molytinae	Hylobiini	*Aclees cribratus*	NC_051548.1	[35]
Cerambycidae*	Lamiinae	Monochamini	*Anoplophora glabripennis*	NC008221.1	[37]
Curculionidae	Molytinae	Cryptorhynchini	*Eucryptorhynchus brandti*	NC_025945.1	[36]
Curculionidae	Molytinae	Pissodini	*Pissodes strobi*	MW452482.1	Unpublished
Curculionidae	Curculioninae	Anthonomini	*Anthonomus eugenii*	NC_044711.1	[45]
Curculionidae	Curculioninae	Anthonomini	*Anthonomus pomorum*	NC_044712.1	[45]

Table S2. Best-fit evolutionary model by mitochondrial gene, as determined on IQ-tree.

Gene	Best-fit model
ATP6	GTR+F+R5
ATP8	TIM2+F+I+G4
COX1	GTR+F+I+G4
COX2	GTR+F+I+G4
COX3	GTR+F+R5
CytB	GTR+F+R6
ND1	GTR+F+R6
ND3	GTR+F+R5
ND4	GTR+F+R6
ND4L	TIM+F+R5
ND5	GTR+F+R6
ND6	TN+F+R5
